# Structure and Stability of Telocentric Chromosomes in Wheat

**DOI:** 10.1371/journal.pone.0137747

**Published:** 2015-09-18

**Authors:** Dal-Hoe Koo, Sunish K. Sehgal, Bernd Friebe, Bikram S. Gill

**Affiliations:** 1 Department of Plant Pathology, Wheat Genetics Resource Center, Throckmorton Plant Sciences Center, Kansas State University, Manhattan, KS, 66506–5502, United States of America; 2 Department of Plant Science, South Dakota State University, Brookings, SD, 57007, United States of America; Leibniz-Institute of Plant Genetics and Crop Plant Research (IPK), GERMANY

## Abstract

In most eukaryotes, centromeres assemble at a single location per chromosome. Naturally occurring telocentric chromosomes (telosomes) with a terminal centromere are rare but do exist. Telosomes arise through misdivision of centromeres in normal chromosomes, and their cytological stability depends on the structure of their kinetochores. The instability of telosomes may be attributed to the relative centromere size and the degree of completeness of their kinetochore. Here we test this hypothesis by analyzing the cytogenetic structure of wheat telosomes. We used a population of 80 telosomes arising from the misdivision of the 21 chromosomes of wheat that have shown stable inheritance over many generations. We analyzed centromere size by probing with the centromere-specific histone H3 variant, CENH3. Comparing the signal intensity for CENH3 between the intact chromosome and derived telosomes showed that the telosomes had approximately half the signal intensity compared to that of normal chromosomes. Immunofluorescence of CENH3 in a wheat stock with 28 telosomes revealed that none of the telosomes received a complete CENH3 domain. Some of the telosomes lacked centromere specific retrotransposons of wheat in the CENH3 domain, indicating that the stability of telosomes depends on the presence of CENH3 chromatin and not on the presence of CRW repeats. In addition to providing evidence for centromere shift, we also observed chromosomal aberrations including inversions and deletions in the short arm telosomes of double ditelosomic 1D and 6D stocks. The role of centromere-flanking, pericentromeric heterochromatin in mitosis is discussed with respect to genome/chromosome integrity.

## Introduction

The centromeres, an essential part of all chromosomes, are responsible for chromosome segregation at mitosis and meiosis. Centromeres usually contain highly repetitive DNA, e.g. satellite DNA, which is associated with proteins in higher eukaryotes [1,2]. Although centromeres are not conserved at the DNA sequence level, many core centromeres analyzed to date contain nucleosomes with a histone H3 variant, CENPA in humans [3] and CENH3 in plants [4]. Thus, these specialized nucleosomes serve as the primary marker of centromere identity. In most eukaryotes, centromere proteins assemble at a single location in each chromosome [5] and are visible as a primary constriction in mitotic metaphase chromosomes. The centromere within a chromosome can move to a new location and form a neocentromere, which is often associated with chromosomal rearrangements in humans [6]. Such neocentromeres also have been reported in plants [7–10].

Each chromosome can be identified based on the centromere position; metacentric, submetacentric, acrocentric, or telocentric [11]. Naturally occurring telocentric chromosomes (hereafter referred as telosomes) are rare in plants. Darlington [12] suggested that the absence of telosomes in plants was caused by their instability. Evidence for the existence of stable telosomes has been provided by Marks [13], Strid [14] and Schubert and Rieger [15] in plants and by Southern [16] and Takagi and Sasaki [17] in animals. According to White [18], experimentally produced telosomes are unstable; and thus, all telosomes are unstable. Experimentally produced telosome stocks in plants, such as wheat (*Triticum aestivum* L.), barley (*Hordeum vulgare* L.), rye (*Secale cereal* L.), and rice (*Oryza sativa* L.), are reported [19,20]. Barley telotrisomics are fairly stable, except for triplo 1L, which shows chimaerism [21]. The question arises, why are some telosomes stable whereas others are not?

Centromeres divide quite regularly at mitosis and meiosis. However, when a chromosome is univalent, centric misdivision may occur during meiosis giving rise to telosomes [12]. Such a chromosome aberration is lethal in diploid organisms but can be tolerated in polyploids. The polyploidy nature of hexaploid wheat, *T*. *aestivum* (2n = 6x = 42), tolerates aneuploidy with either the addition or deletion of chromosomes. Sears and Sears [19] developed several aueuploid stocks in Chinese Spring (CS) wheat including ditelosomic stocks (one chromosome pair is substituted by a pair of either short or long arm telosomes, 2n = 40+2t; such stocks are nullisomic for one of the arms) and double ditelocentric stocks (one chromosome pair is substituted by a pair of short and long arm telosomes, 2n = 40+4t; such stocks are euploid). These stocks were used intensively for centromere mapping and allocating genes and markers to specific chromosome arms [22,23]. Moreover, the telosomes in the ditelosomic (hereafter Dt) and double ditelosomic (hereafter dDt) stocks are about half the size of a metacentric chromosome, making them amenable for flow sorting [24–26].

Flow-sorted telosomes are the foundation material for chromosome-arm-based BAC libraries and the wheat physical maps developed under the auspices of the International Wheat Genome Sequencing Consortium project [27]. Recently, individual flow-sorted chromosome arms were used to generate a draft sequence of the 17-Gb wheat genome [28]. Even with the extensive use of telosomic stocks for genetic and genomic studies in wheat, their detailed cytogenetic nature is poorly understood. The cytological stability of a telosome depends on the structure of its kinetochore. Steinitz-Sears [29] reported that the relative instability of a telosome may be attributed to the degree of completeness of its kinetochore. Because wheat telosomic stocks were developed by centric misdivision and stably transmitted to progeny, they are supposed to have either complete or nearly complete kinetochores.

In this study, we first developed a wheat CENH3 antibody (see experimental procedure) and then used it to identify and characterize the functional centromeric region of the telosomes. Second, centromeric-specific retrotransposons of wheat (CRWs) [30,31] were used to study the structure of intact and telosomic chromosomes. D-genome-specific, repetitive DNA (pAs1) [32] and single-copy DNA probes [33] were used to identify chromosomes and characterize chromosomal rearrangements. In addition, chromosome-arm-specific molecular markers, derived from the wheat deletion bin map (http://probes.pw.usda.gov:8080/snpworld/Map) [34], were used to detect chromosomal aberrations. The data provide new insights into the structure and stability of telocentric chromosomes and their centromeres; the implications of these results to genetic and genomic studies of wheat are discussed.

## Materials and Methods

### Plant material and chromosome preparation

The cytogenetic stocks used in this study are listed (Table 1). Wheat cultivars, Chinese Spring (CS), Jagger, and TAM111, also were used in molecular and cytogenetical studies. For chromosome preparations, seeds were germinated in petri dishes on moist filter paper. Root tips (1–2 cm long) were treated overnight in ice water. The root tips were fixed overnight in a 3:1 ethanol:glacial acetic acid and then squashed in a drop of 45% acetic acid. For the immunofluorescence of CENH3, ice-cold-treated root tips were fixed immediately using 4% paraformaldehyde in PHEM (60 mM Pipes, 25 mM Hepes, 10 mM EGTA, 2 mM MgCl_2_, and 0.3 mM sorbitol, pH 6.8) for 40 min. After washing with 1x PBS (10 mM sodium phosphate, pH 7.0, and 140 mM NaCl), the root tips were treated with 2% cellulase, 1% pectinase (Sigma, St. Louis, MO) and 1% pectolyase in PHEM for 1 h and then squashed on poly-l-lysine coated slides (Sigma). All preparations were stored at -70°C until use.

**Table 1:** Wheat cytogenetic stocks used in this study.

Cytogenetic stock	Source/Plant ID
Dt1DS	WGRC, TA3087
Dt1DL	WGRC, TA3131
dDt1D	WGRC, TA3158
Dt2DS	WGRC, TA3123
Dt2DL	WGRC, TA3124
dDt2D	WGRC, TA3146
Dt3DS	WGRC, TA3193
Dt3DL	WGRC, TA3192
dDt3D	WGRC, TA3147
Dt4DS	WGRC, TA3125
Dt4DL	WGRC, TA3126
dDt4D	WGRC, TA3148
Dt5DS·Mt5DL	U.C Riverside
Dt5DL	WGRC, TA3127
dDt5D	WGRC, TA3149
Dt6DS	WGRC, TA3128
Dt6DL	WGRC, TA3129
dDt6D	WGRC, TA3150
Dt7DS	WGRC, TA3130
Dt7DL	WGRC, TA3071
dDt7D	WGRC, TA3151
dDt1A	WGRC, TA3132
dDt2A	WGRC, TA3133
dDt3A	WGRC, TA3134
dDt5A	WGRC, TA3136
dDt6A	WGRC, TA3137
dDt7A	WGRC, TA3138
CS dDt1B-dDt2B-dDt3B-dDt4A-dDt5B-dDt6B-dDt7B	WGRC, TA3356

Dt: ditelosome, one chromosome is represented by a pair of either short or long arm telosomes (2n = 40+2t). dDt: double ditelosome, one chromosome is represented by one pair each of short (S) and long (L) arm telosomes (2n = 40+4t).

### Immuno-detection of CENH3 and fluorescence *in situ* hybridization (FISH)

Wheat *CENH3* genes were described previously [31]. A peptide antigen, ‘RTKHPAVRKTKALPKK’, was synthesized and used to immunize rabbits at Thermo Fisher Scientific (www.thermofisher.com). The raised antisera were purified using an affinity sepharose column comprising the aforementioned peptide. The specificity of the antibody was checked by immunostaining of root tip and pollen mother cells of wheat (data not shown). Immuno-detection of CENH3 and FISH procedures followed previously published protocols [35–37]. The rabbit antibodies to CENH3 were diluted to 1:1000 in TNB buffer (0.1 M Tris-HCl, pH 7.5, 0.15 M NaCl, and 0.5% blocking reagent). Approximately 100 μL of the diluted antibodies was added to each slide, and the slides were incubated in a humid chamber at 37°C for 2–3 h. After three washes in 1x PBS, 100 μL of rhodamine-conjugated goat anti-rabbit antibody (Jackson ImmunoResearch, West Crove, PA) (1:100 in TNB buffer) was added to the slides. Incubation and washes were the same as for the primary antibody. DNA probes of the CRWs, pAs1, pSc119, and the other single-gene probes were labeled with digoxigenin-11-dUTP, biotin-16-dUTP, and/or DNP-11-dUTP, depending on whether two or three probes were used in the FISH experiment. The cDNA clones used in this study were supplied by the National BioResource Project-Wheat, Japan. After post-hybridization washes, the probes were detected with Alexafluor 488 streptavidin for biotin-labeled probes, and rhodamine-conjugated anti-digoxigenin for dig-labeled probe. The DNP-labeled probe was detected with rabbit anti-DNP, followed by amplification with a chicken anti-rabbit Alexafluor 647 antibody.

Multicolor immuno-FISH detection was described previously [36]. Chromosomes were counterstained with 4′,6-diamidino-2-phenylindole (DAPI) in Vectashield antifade solution (Vector Laboratories, Burlingame, CA). The images were captured with a Zeiss Axioplan 2 microscope (Carl Zeiss Microscopy LLC, Thornwood, NY) using a cooled CCD camera CoolSNAP HQ2 (Photometrics, Tucson, AZ) and AxioVision 4.8 software. The final contrast of the images was processed using Adobe Photoshop CS5 software.

### Sequential detection of CENH3, CRWs, pSc119 and pAs1

For sequential detection the slides were first incubated with anti-CENH3 overnight at 4°C in a wet chamber. After washes in 1x PBS, the slides were incubated with the appropriate secondary antibody at 37°C for 50 min. Then slides were re-fixed with 4% paraformaldehyde at RT for 30 min. The slides were then denatured in 70% formamide in 2x SSC, 80°C for 2 min, washed in ice-cold 1x PBS for 5 min, and then DNA probe, CRWs, was applied to the slides. Post-hybridization wash and signal detection were the same as FISH procedure. After recording the both CENH3 and CRWs signals, the slides were washed in 4T (4x SSC/0.05% Tween 20) buffer for 1 h at 37°C and re-fixed with 4% paraformaldehyde and dehydrated in an ethanol series. The slides were re-probed with pAs1 and pSc119 to detect additional sequences on the same chromosome.

### Genome-specific markers and PCR

The genome-specific primers used are listed in Table 2. PCR was performed with 15 μL of the reaction mixture containing 1x PCR buffer (Bioline USA Inc., Taunton, MA), 2 mM MgCl2, 0.25 mM dNTPs, 5 pmol forward primer and reverse primer, respectively, 0.02 U/μL Taq DNA polymerase (Bioline USA Inc., Taunton, MA), and 20 ng genomic DNA. PCR amplification was according to Liu et al. [38]. PCR products were resolved on 2.5% agarose gels and visualized by ethidium bromide staining under UV light.

**Table 2:** Wheat genome specific EST markers used in this study.

EST marker	Forward primer	Reverse primer	Deletion bin
BE405518	GTCTCAGGTATTGATTGATCCC	GCTGATGCTCCTTGATCTCC	1DS0.70–1.00
BE637971	TGCCTGATGTTTGATGCTCC	CAAAGCGAAGTGACTGTCCA	1DS0.70–1.00
BE444846	TCTTCGCCACAGGAGTACCTA	GGCTCGTAGCGGGTATACAA	1DS0.00–0.48
BE591601	GTTAGTGGCACTCCTACCTG	GATGTCCAACCATAATGCCC	1DS0.00–0.48
BE637864	TCCTCATTTTGTAATCCTTCTCTC	TTTTGTTCCCACCATCAGGT	1DS0.00–0.48
BF202643	GAATAGCAACAGTGCTCATGAAT	GAAGAACAGCAGGGCGTTAC	1DS0.00–0.48
BF474569	CGTACCAACTCAACCCCTC	TGAAGGGTGAGAGAACTCCG	1DS0.00–0.48
BF478737	CTCTTCACAGTTACAACATCAGC	TGAGGCTCAATGATGACCAG	1DS0.00–0.48
BE424523	CAGTAAGGAAATATGGCCGAT	TTGATGCAGAAAAAGTTGGAT	6DS0.79–0.99
BE490604	AAGCGGTTCCATCTCTCC	CTGCCATTGCTTGTCGTAGA	6DS0.79–0.99
BE500768	ACCTCGACCACTCACTCCA	TCAGCGGTCTCAGTTTGTTG	6DS0.79–0.99
BE517858	CCGGTGATGACCGAACTGAT	CCGGATGATCTCGCTGCTCTC	6DS0.79–0.99
BE444631	CTCCAGTTTCAGGGAGCAAG	GTTCTCTGGCAAGTACTTCAAATCC	6DS0.45–0.79
BE445201	AATGAATTGCTACCATTATTCTCA	ACAGCCGTGAACGTTAGTAAGT	6DS0.45–0.79
BF478958	TCACCTGTACAACAACATGATTTCAA	TGTGCTCATATGTTTTAACT	6DS0.45–0.79
BF483025	ATTCTGTAAGCATGACGGC	AAGGAACTAAGGCCAAGCAATT	6DS0.45–0.79
BE405809	AACGATGCAAGGCTAAAATCTGTGT	GAAGCTGCTGGTTTCTTTGG	6DS0.00–0.45
BE426591	CAGACAATCTTCTTGCCGCT	GTTAGAAATACCGTAAAGCTTTTACCATTAC	6DS0.00–0.45

## Results

**Fig 1:**
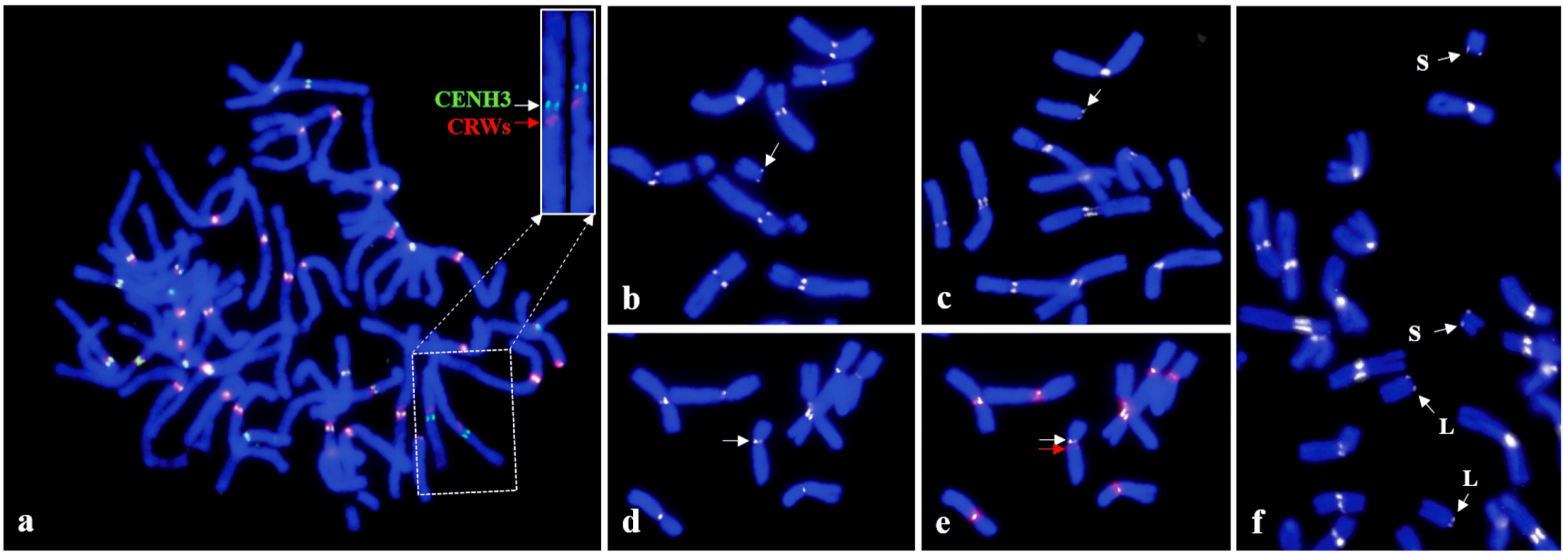
Detection of CENH3 and CRWs on mitotic chromosome of Chinese Spring (CS) wheat and the derived 4D telosomic stocks; a: the insert shows chromosome 4D probed with CENH3 (green) and CRWs (red), straightening was performed using the ‘straighten-curved-objects’ command of the Image J software, the signals are clearly separated from each other; Relative CENH3 signal intensity of b: t4DS; c: t4DL; f. dDt4D; Chromosome 4D with d: CENH3 (white arrow) and e: CRWs (red arrow) signals.

Immunofluorescence of CENH3, coupled with centromeric DNAs (CRWs), was used to identify the centromeric regions of intact chromosomes and telosomes of wheat. The localization pattern of CENH3 and the CRWs on mitotic metaphase chromosome of CS and their derived telosomes is shown in Fig 1. Consistent with previous reports [30,31], the two probes co-localized in most chromosomes of CS wheat. However, in one chromosome pair of CS, identified as 4D, the two probes were clearly separate from each other (Fig 1a), suggesting that not all CRWs are located within the functional centromere. Interestingly, in the two winter wheat cultivars Jagger and TAM111, the two signals co-localized on chromosome 4D, indicating that the 4D centromeres in CS underwent repositioning (S1 Fig).

Next, we applied the wheat CENH3 antibodies to study the structure of t4DS (telosome for 4DS arm) and t4DL, which arose independently from centric misdivison of chromosome 4D. The results showed that CENH3 was detected at the extreme ends of t4DS and t4DL, indicating that they are true telosomes (Fig 1b and 1c). The immunofluorescence of CENH3 on the telosomes was weaker compared to that of the other chromosomes. In order to compare the signal intensity between the intact chromosome 4D and t4DS and to minimize measurement error, root tips from euploid CS and the t4DS stock were squashed on the same slide, and chromosome images probed with CENH3 were captured from the same preparation. The measurement data showed that t4DS had a signal intensity of 43±4.8% (n = 4), compared with that of an intact 4D chromosome. To compare the signal intensity between t4DS and t4DL, we used the dDt4D stock, which contains a pair each of t4DS and t4DL (Fig 1f). The result showed that both telosomes had a similar amount of signal intensity, approximately a 1:1 ratio (79.6±3.8% in t4DS: 80.2±1.7% in t4DL, n = 4) (S2 Fig). Although the dDt4D stock was developed by intercrossing the t4DS and t4DL telosomes, which arose independently from centric misdivison, they showed approximately half of the signal intensity compared with that of the intact 4D chromosome.

**Fig 2:**
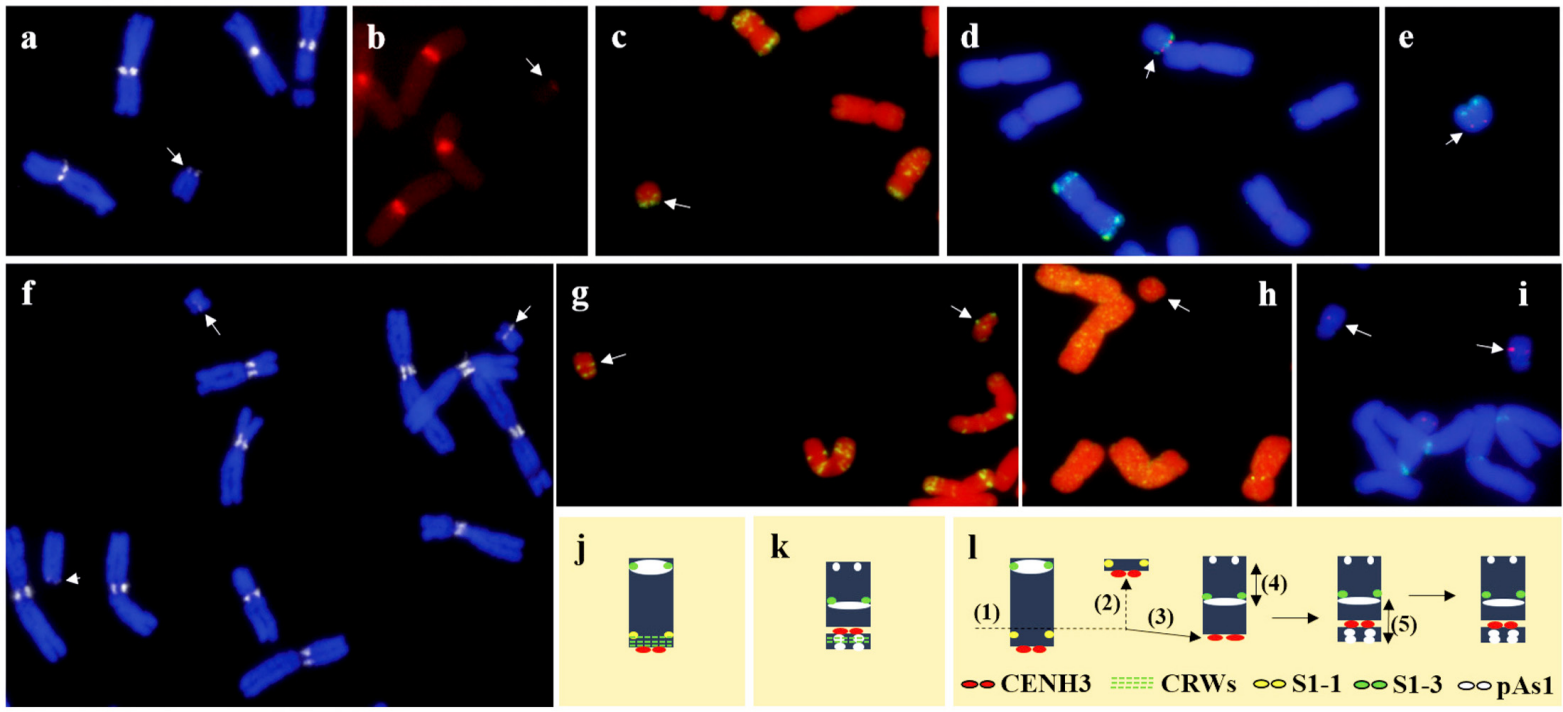
Probing of t1DS telosomes present in the Dt1DS stock with a: CENH3, b: CRWs, c: pAs1, d: 1S-3 (red dots) and pAs1 (green), e: 1S-1 (red dots) and pAs1 (green); Probing of 1DS telosomes present in the dDt1DS stock with f: CENH3, g: pAs1, h: 1S-1 (not detected), and i: 1S-3 (red dots) and CRWs (faint green signals). Simultaneous detection of CENH3, CRWs and pAs1 on dDt1DS also provided in S3 Fig. Ideograms depicting localization of each probe on telosomes, Dt1DS and dDt1DS (j-k, and S3 Fig). Possible scenario of the origin of the chromosomal rearrangements observed in the dDt1DS: (1) chromatin breakage, (2) loss of original centromere, (3) *de novo* formation of a centromere, (4) paracentric inversion, (5) pericentric inversion (s).

**Fig 3:**
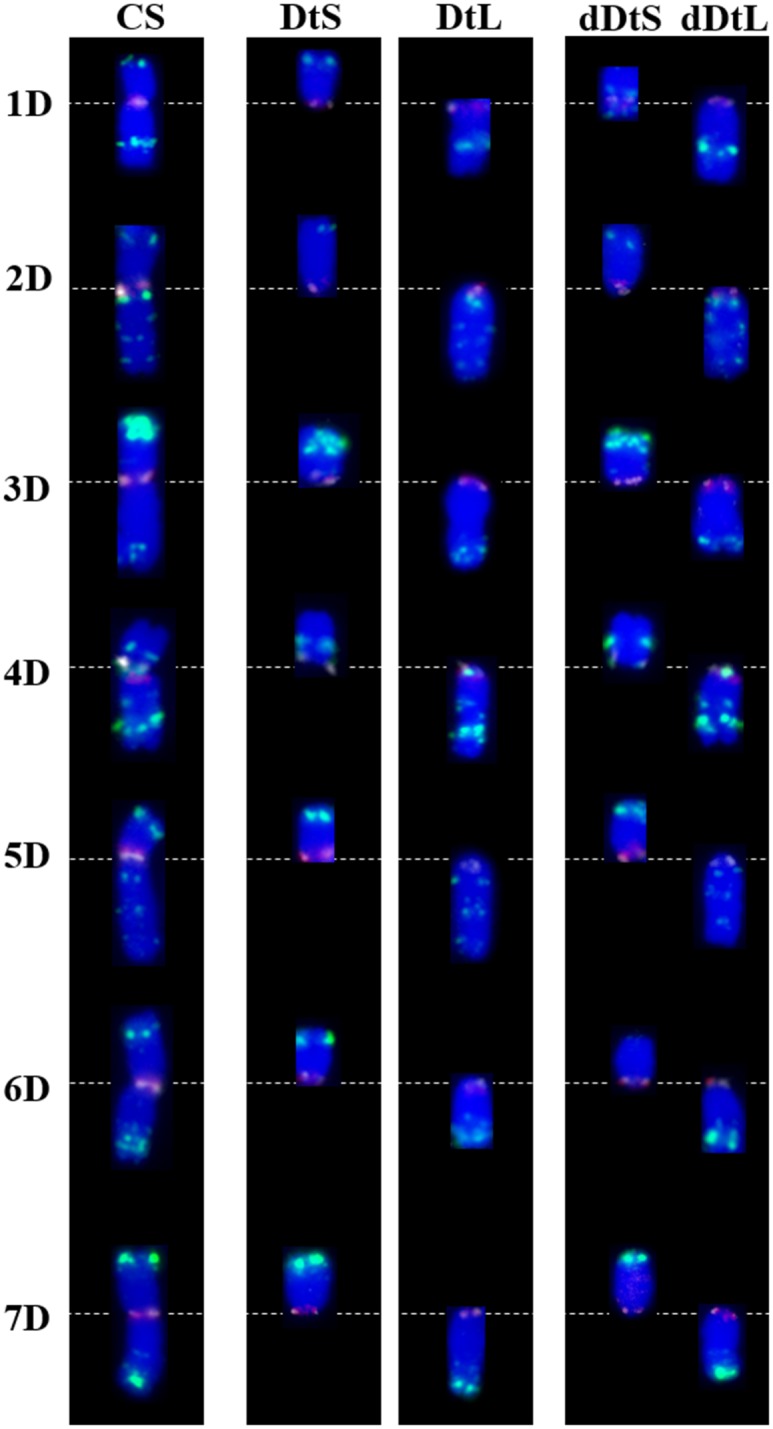
Immuno-FISH based karyotype of D-genome chromosomes of wheat and their derived telosomes using CENH3 (white), CRWs (red) and pAs1 (green) as probes. CRWs (red signals) co-localized with CENH3 (white signals) in most of the chromosome except dDt1DS, 4D, Dt4DS, dDt4DS, Dt5DL and dDt5DL. The centromeric regions of chromosome or chromosome arm were seen as pinkish red colors because the CRWs (red signals) are abundant in centromeric region and much brighter than CENH3 signals except in the above mentioned telosomes. The dDt1DS stock contained multiple chromosome rearrangement including inversion, deletion and centromere shift. Note that the CRWs were not detected in Dt4DS and dDt4DS, instead the pAs1 signal was overlapped with the CENH3 signal in these telosomes. A very faint pAs1 FISH site was detected in the terminal region of dDt6DS, indicating a terminal deletion. Short arm and long arm telosomes present in the ditelosomic stocks are represented as (DtS) and (DtL), respectively and short arm and long arm telosomes present in the double ditelosomic stocks are represented as (dDtS) and (dDtL), respectively.

We also studied the telosomic derivatives of the seven, D-genome chromosomes of wheat (Figs 2 and 3). Data on the detection of CENH3, the CRWs, pAs1, and single-copy DNA probes on Dt1DS and dDt1DS are shown (Fig 2 and S3 Fig). Signals for CENH3 (arrow in Fig 2a) and the CRWs (arrow in Fig 2b) on Dt1DS were located at the end of the chromosome, indicating that it is a true telosome. In the t1DS of the reconstituted dDt1D stock, however, CENH3 was localized interstitially, forming a small acrocentric chromosome (arrows in Fig 2f). To further discern the chromosomal rearrangement, we performed FISH using pAs1, 1S-1, and 1S-3 as probes. Hybridization signals for pAs1 (arrows in Fig 2c and 2d) and 1S-3 (red dots in Fig 2d) were detected on the terminal region of Dt1DS. However, the pAs1 FISH pattern in dDt1DS showed multiple localizations (arrows in Fig 2g) to both telomeric regions and the interstitial region of the chromosome arm. Probe 1S-3 (arrows in Fig 2i) was detected in the middle of the arm, instead of in the telomeric region, indicating the presence of a paracentric inversion (Fig 2l). Moreover, the FISH signal for 1S-1 (arrow in Fig 2h) was not detected in the pericentromeric region of dDt1DS, implying that dDt1DS has lost the original centromere and now has a *de novo* centromere in a new position (Fig 2l). Supporting evidence came from PCR analysis using six genome-specific markers derived from the 1D proximal bin, which failed to amplify in the dDt1D stock but did amplify in CS and Dt1DS (S4 Fig). Thus, dDt1DS contains multiple chromosomal rearrangements, including a centromere shift, a paracentric inversion, and a deletion. The labeling patterns of CENH3, the CRWs, and pAs1 on dDt1DL were similar with those of Dt1DL (Fig 3).

The same approach was used to analyze all the D-genome telosomes, and the results are presented (Fig 3). The FISH pattern of pAs1 on chromosome 2D showed multiple localizations, with four FISH sites in the long arm and a single hybridization site on the telomere of the short arm. CENH3 and the CRWs co-localized on the primary constriction (Fig 3). Applying these probes to the Dt and dDt stocks revealed largely identical hybridization patterns with those of an intact 2D chromosome, indicating that there are no rearrangements in these telosomes. Similar patterns were observed for all the remaining D-genome telosome stocks, except for chromosome 6D.

**Fig 4:**
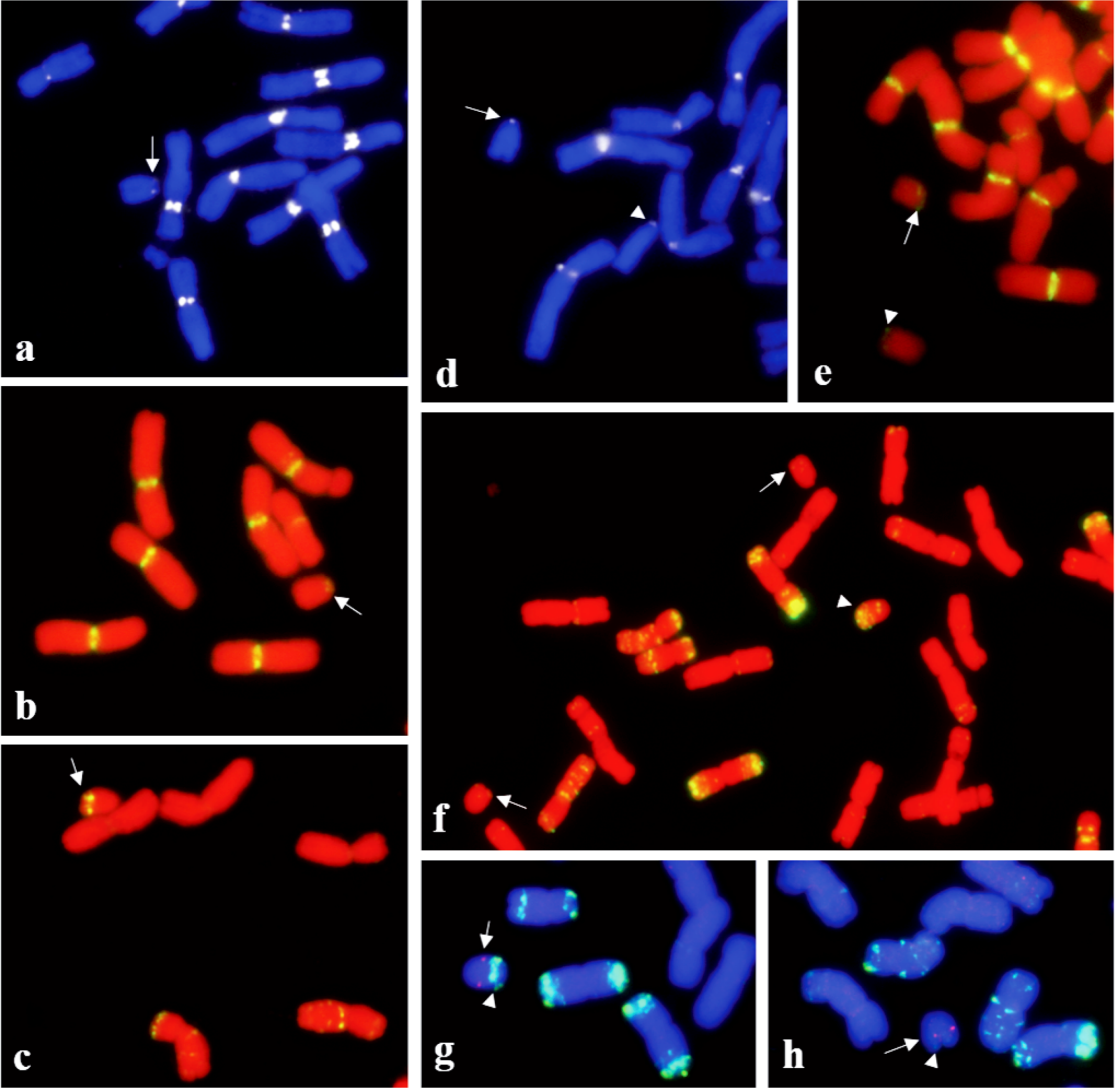
Localization of CENH3 (arrow in a), CRWs (arrow in b) and pAs1 (arrow in c) on Dt6DS; localization of CENH3 (d), CRWs (e) and pAs1 (f) on dDt6D, arrows and arrowhead indicate the 6DS and 6DL telosomes, respectively. Two color detection of single gene probe, 6S-2 and pAs1 on Dt6DS (g) and dDt6DS (h); the hybridization signal for single copy probe 6S-2 (red dots) is indicated by arrows and the pAs1 was labeled with green colors by arrowheads.

CENH3 and the CRWs were detected at the end of chromosome arms in Dt6DS (arrows in Fig 4a and 4b) and dDt6DS (arrows in Fig 4d and 4e). Whereas a prominent pAs1 signal was located on the subtelomeric region of Dt6DS (arrows in Fig 4c and 4g), a faint hybridization signal was detected on dDt6DS (Fig 4f and 4h) indicating that the chromosome arm of dDt6DS has suffered from a terminal deletion. The 6S-2 FISH signal on Dt6DS was detected at 61.0±3.1% (n = 3) from the telomere (Fig 4g). However, in dDt6DS, the 6S-2 FISH signal was detected 39.9±3.2% (n = 3) from the telomere (Fig 4h), thus about 20% of the distal region was deleted. In order to verify the deletion at the molecular level, we used genome-specific PCR primers, which were derived from the terminal deletion bin of 6DS (S5 Fig). Four markers derived from terminal bin had no amplification; the other six markers derived from interstitial and proximal bins had amplification (S5 Fig), confirming that about the distal 20% of the telosome was deleted. We made a blastn search of ten EST sequences against the sequence assembly from flow-sorted chromosome arm 6DS [28] and found no hit for markers *XBE424523*, *XBE490604*, *XBE500768*, and *XBE517858* derived from terminal deletion bin. We further analyzed the genome zipper maps of wheat group 6 [28] and observed that this region is deleted in t6DS, whereas the corresponding region is present in t6AS and t6BS. This region corresponds to rice locus *Os02g0116800*-*Os02g0128800* [39], which is about 595 kb in rice and 702 kb in *Brachypodium Bradi3g01540*.*1-Bradi3g02817*.*1* [40]. This region is syntenic with the terminal tip of rice chromosome 2. Nearly 100 genes are annotated in this missing region in the rice genome, 25 of which are syntenic to wheat. Thus, sequence analysis further confirms the loss of a segment from dDt6DS. Because the available dDt1D and dDt6D stocks are rearranged, we re-isolated both stocks, which are now intact and similar to the Dt1DS and Dt6DS stocks. The labeling patterns of CENH3 in the new stocks were similar to those of the DtS lines (data not shown). Cytogenetic analysis is in progress to investigate the mitotic behavior on the newly developed dDt stocks.

To further understand the CENH3 deposition on other telosomes, we used dDt lines that were derived from all the A- and B-genome chromosomes. Our results on immunofluorescence of CENH3 in dDt lines derived from A-genome chromosome showed that the position of CENH3 signals on telosomes is terminal with a signal intensity weaker than that of the other regular chromosomes (S6 Fig). Consistent with dDt4D, signal intensity for CENH3 between the DtS and DtL arms in dDt lines showed similar intensity except in dDt4AS (arrowheads in S6 Fig). The telosome, dDt4AS is known to be acrocentric and, thus, contains a complete centromere [41].

**Fig 5:**
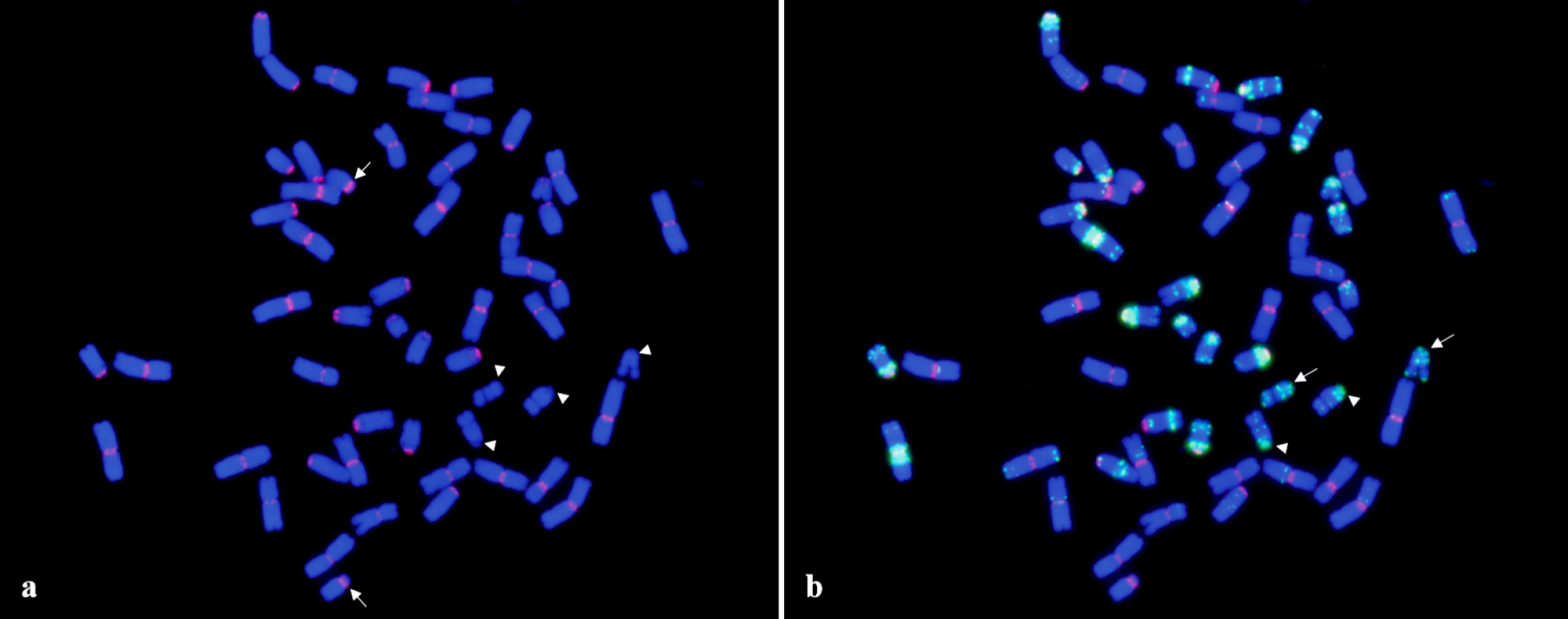
Two color FISH mapping of CRWs (red) and GAAn (green) repeats on mitotic metaphase chromosome of CS containing 28 (24 telosomes from B-genome telosomes and 4 telosomes from t4AS and t4AL). The signals for CRWs were not detected on two pairs of t1BS and t6BS (arrowheads) (a). Instead GAAn repeats were presented in their centromeric region. Arrows indicate the t4AS (a). Arrows and arrowheads indicate the t1BS and t6BS, respectively (b).

To study the B-genome telosomes, we used a line that has all the B-genome chromosome arms except 4B as telosomes (2n = 28+28t) [42]. This line was obtained by intercrossing the appropriate dDt stocks. Chromosomes t4BS and t4BL in this stock were later identified as t4AS and t4AL telosomes [43]. Likewise, we observed that the CENH3 signal on all B-genome telosomes was smaller compared with that in the complete chromosomes (S6 Fig). In addition, the telosomes, including Dt1BS and Dt6BS in this stock, lack CRWs in the CENH3 region (Fig 5), indicating that the stability of telocentric chromosomes depends on the presence of CENH3 chromatin but not centromeric DNA repeats. Further analysis using the previously known wheat centromeric repeats CCS1 [44], the 192-bp repeat [45], and Quinta [31] showed no signal on these telosomes together with Dt4DS, dDt4DS, Dt5DL, and dDt5DL (data not shown), further indicating that centromeric DNAs are not essential for normal centromere function.

## Discussion

In many plant species, centromeres consist of complex DNA, including satellite DNA and reterotransposons [2], that are species- and chromosome-specific [46,47]. Some of the centromeric repeats are located within the functional centromere, but centromere-associated repeats also are observed in subtelomeric and interstitial chromosome regions [46,48].

Our study, using immuno-FISH probed with CENH3 and CRWs, shows that CENH3 and CRW elements co-localized on most of the A-, B- and D-genome chromosomes, including their telosomic derivatives. These results indicate that wheat centromeres contain CRW elements that interact with wheat CENH3 [30,31]. However, our results further show that wheat CRWs do not always co-localize with CENH3, as was the case with the complete chromosome 4D and some of the telosomes, including Dt4DS, dDt4DS, Dt5DL, dDt5DL, Dt1BS, and Dt6BS, because these chromosome or chromosome arms lack CRWs in their CENH3.

The CRWs located on chromosome 4D in CS did not overlap with the CENH3 signals but in other wheat cultivars, such as Jagger and TAM111, they co-localized in the same region, providing evidence for centromere repositioning in chromosome 4D of CS (S1 Fig). Comparison of the pAs1 FISH pattern in the pericentromeric region of chromosomes 4D between CS, Jagger, and TAM111 revealed that their pericentromeric localizations were different from each other, implying that the pericentromeric region of 4D in CS underwent a structural rearrangement. The pAs1 FISH site overlapped with CENH3 in CS but not in Jagger or TAM111 (S1 Fig). Lo et al. [49] and Lomiento et al. [50] have reported that neocentromeres form in gene-desert regions containing many repetitive DNAs in many species. These results also indicate that neocentromere function is independent from the presence of original centromeric DNAs [51]. Our immuno-FISH results on telosomes also support their stabilization without centromeric DNAs, because we did not observe CRW signals in Dt1BS and Dt6BS arms. In these telosomes, the GAA repeats co-localized with CENH3 (Fig 5). Thus, wheat centromeres consist of complex DNAs, which may contain CRWs or satellite DNAs, such as pAs1 and GAAn.

Univalent chromosomes at metaphase-I have the tendency to misdivide at the centromere in a transverse manner, which gives rise to telocentrics or potential isochromosomes. Steinitz-Sears [29], suggested that the transverse misdivision split can occur in different regions of the centromere, resulting in telosomes that differ in the completeness of their centromeric regions, and that chromosome arms with incomplete or partial centromeres behave like acentric fragments and are lost during cell division. Because the set of wheat telosomes was produced by centric misdivision and they are stably transmitted to the offspring, they must have received a complete or nearly complete kinetochore. Similarly, barley and rye telocentric chromosomes are cytologically stable. Giemsa N-banding on barley telotrisomics revealed that they contain half of a diamond-shaped kinetochore, whereas complete chromosomes contain an intact, diamond-shaped kinetochore [52]. Rice telocentric chromosomes also contain half of CentO, compared with its normal centromeres [53].

Our immunostaining analysis using the CENH3 antibody suggests that the signals in most Dt and dDt telosomes had approximately half or even less the CENH3 signal intensity compared with that of a complete chromosome. None of the derived telosomes received a complete CENH3 region except dDt4AS, which is an acrocentric chromosome (S6 Fig). These results indicate that telosomes, which only receive half or less than half of the complete CENH3 region, are cytologically stable and transmitted to the offspring. Because most of the wheat telosomes received a partial CENH3 region, it is possible that the transverse misdivision split is not random and may preferentially occur in the middle of the CENH3 chromatin. However, we cannot rule out that breakage also occurs in the entire functional centromere region and that telocentric chromosomes with insufficient CENH3 are mitotically unstable and lost. This is also supported by analyzing the centromere structure of wheat-rye Robertsonian translocations derived from repeated centric breakage-fusion events, which revealed that breakage can occur in different regions of the centromere resulting in wheat-rye hybrid centromeres with different sizes of wheat and rye centromeric repeats [54].

We observed chromosomal aberrations in dDt1DS and dDt6DS. Double labeling of CENH3 and CRWs on the Dt1DS telosomes showed co-localization at the terminal region. In dDt1DS, however, a very faint CRW hybridization signal was observed at the telomere, and CENH3 was localized proximal to it. Single-copy, 1S-1 FISH was not detected in the proximal region of dDt1DS, indicating the presence of a proximal deletion and supporting a previous finding that ESTs mapped in the wheat 1DS deletion bin were absent in dDt1DS [55]. The deletion placed the centromere in a new position. Thus, dDt1DS contains a *de novo* formed centromere (Fig 2l).

The formation of a *de novo* centromere in dDt1DS supports earlier reports in *Drosophila* [56] and chicken (*Gallus gallus*) [57], where neocentromeres formed in regions close to the original centromeres. For instance, when the Z centromere was deleted, neocentromeres most frequently formed near the original Z centromere [57]. CENP-A/CENH3 enrichment in the flanking regions is low but still more enriched compared with that in the rest of the genome [58]. In our study, the formation of *de novo* centromeres or centromere shift was observed in chromosome 4D and in the 1DS telosomes present in the dDt1DS stock. The preference of the formation of the *de novo* centromere near the original centromere is likely caused by the presence of CENH3 in the flanking pericentromeric regions. Chromosome rearrangements after chromatid breaks are a common cause of neocentromere formation in humans [6]. Likewise, neocentromeres reported in plants, maize chromosomes in an oat background and barley chromosomes, also were associated with loss of endogenous centromeres by chromatid breakages [7,8]. We also found a paracentric inversion in telosome dDt1DS using single-copy FISH mapping. Interestingly, the pAs1 signal was observed near the *de novo* centromere region and in an interstitial region of dDt1DS but was absent in Dt1DS, indicating the possibility of another chromosome rearrangement (Fig 2l).

We also identified a deletion in dDt6DS, which comprised about 20% of the terminal deletion bin of chromosome 6D. This deletion was not reported in a recent whole-genome, sequencing analysis [28]. However, our results show that several EST sequences from the terminal deletion bin were missing from the 6DS assembly but present in 6AS and 6BS shotgun sequence assemblies [28]. Thus, when employing these lines for genetic studies, it is important to be aware of the potential presence of chromosomal aberrations in the telocentric chromosome lines to avoid misinterpretation of experimental results. Further relocation of centromeres in chromosomes 4D in CS (the cultivar being used to sequence the wheat genome) compared to other wheat cultivars, Jagger and TAM111, suggests that *de novo* sequencing of more wheat genotypes might provide further insight in the structural organization in the wheat genome.

The wheat dDt stocks were developed by intercrossing the appropriate Dt stocks followed by selection. Therefore, chromosomal rearrangements, including centromere shifts, deletions, and inversions observed in dDt1DS and dDt6DS, might have formed after hybridization of the Dt lines. Rhoades [59] found that telocentric chromosomes in maize undergo structural changes during somatic cell divisions leading to loss or diminution in size. Steinitz-Sears [29] also found that a telocentric chromosome is often unstable and may be lost during plant development in wheat. During mitosis, the structural integrity of the centromeric and flanking pericentric heterochromatic regions is essential for proper assembly of the kinetochore and genome stability [60]. In fission yeast, pericentromeric heterochromatin is an absolute requirement for the establishment of the centromere [61]. In addition to fission yeast, pericentromeric heterochromatin seems to be required for the accurate segregation of chromosome during mitosis in many eukaryotes, including mammals [62]. The implication is that mono-arm oriented pericentromeric heterochromatin in telosomes might be relatively insufficient for maintaining chromosome stability compared to chromosomes with bi-arms oriented pericentromeric heterochromatin. This conclusion is supported by recent findings of Wanner et al. [63] that in monocentrics microtubules attach via CENH3 to both pericentromeres to stabilize the chromosomes during anaphase against the pulling forces.

## Supporting Information

S1 Fig Sequential detection of CENH3, CRWs, pSc119 and pAs1 on the chromosomes of 4D in CS (A), Jagger (B) and TAM111 (C). The hybridization signals for CENH3 and CRWs were clearly separated from each other in 4D of CS but these were co-localized on the chromosomes of 4D in Jagger and TAM11, indicating the centromere repositioning in 4D of CS. In 4D of CS, pAs1 localization pattern tend to be positioned toward log arm and which is completely overlapped with CENH3. In Jagger and TAM111, however it was positioned toward to the short arm. The pSc119 was used for additional FISH marker to identify the chromosomes 4D in CS, Jagger and TAM111. D, Ideogram depicting distribution of each probe on the chromosomes of 4D in three wheat cultivars.
(TIF)

S2 Fig Graph showing the measurements of the immunofluorescence signal intensity of CENH3. Numbers at y axis represent the gray value (relative signal intensity of antibody to background, background was normalized as zero). 1: background signal, 2: CENH3 signal intensity in dDt4DS, 3: CENH3 signal intensity in dDt4DL. Measurements were done by Image J software. The gray value of CENH3 was 79.6±3.8 (n = 4) and 80.2±1.7 (n = 4) in dDt4DS and dDt4DL, respectively.
(TIF)

S3 Fig Multicolor immuno-FISH detection of CENH3 (a), CRWs (b) and pAs1 (d) on telosome, dDt1D. Merged images, CENH3 and CRWs (c), and CENH3, CRWs and pAs1 (e) with DAPI stained metaphase chromosome (f). The inserts show telosome, dDt1DS probed with CENH3 (red), CRWs (green) and pAs1 (white). CENH3 was detected by rhodamine-conjugated anti-rabbit antibodies (red), and the signals were fixed with 4% paraformaldehyde. The same metaphase cell was probed with CRWs (green) and pAs1 (far red, the signals were pseudocolored in white).
(TIF)

S4 Fig PCR patterns of CS, Dt1DS, Dt1DL and dDt1D by using genome specific primers: two primers, BE405518 and BE637971, derived from the terminal deletion bin, BE405518 was not amplified (2); six primers, BE444846, BE591601, BE637864, BF202643, BF474569 and BF478737, derived from proximal bin had no amplification (3–8) indicating proximal deletion in dDt1DS. (TIF)

S5 Fig PCR patterns of CS, Dt6DS, Dt6DL and dDt6D by using genome specific primers: four primers, BE424523, BE490604, BE500768 and BE517858 derived from the terminal deletion bin; four primers, BE444631, BE445201, BF478958 and BF483025 derived from interstitial bin; two primers, BE405809 and BE426591 derived from proximal bin. Four primers derived from terminal deletion bin had no amplification (1–4) while six primers derived from interstitial (5–8) and proximal deletion bin (9–10) had amplification in dDt6DS indicating terminal deletion in dDt6DS.
(TIF)

S6 Fig Partial metaphase cells probed with CENH3 in dDt lines derived from A-genome chromosomes: a, dDt1A; b, dDt2A; c, dDt3A; d, dDt5A; e, dDt6A; f, dDt7A. Telosomes are indicated by arrows. Immunofluorescence of CENH3 on CS containing 28 telosomes (g). The CENH3 fluorescent signals on 24 telosomes (derived from B-genome chromosomes are indicated by arrows) + one pair of t4AL were smaller than those of other intact chromosomes except 4AS (arrowhead) which is an acrocentric chromosome.
(TIF)
