# Supplementary figures and images for: Structure and Stability of Telocentric Chromosomes in Wheat
Source: PLoS One. 2015 Sep 18;10(9):e0137747. doi: 10.1371/journal.pone.0137747 (PMC4575054; doi:10.1371/journal.pone.0137747)

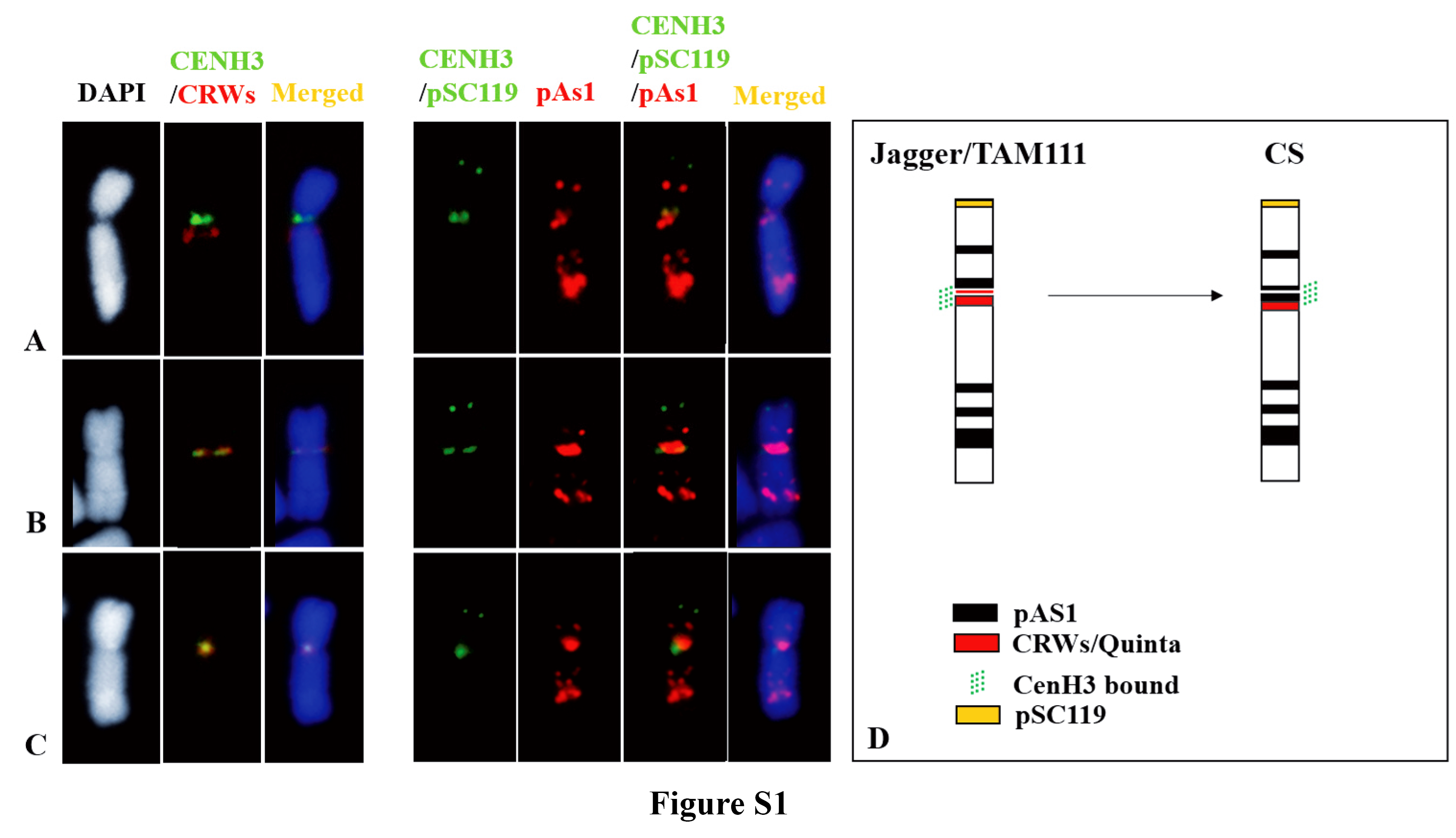

Supplement: S1 Fig — The hybridization signals for CENH3 and CRWs were clearly separated from each other in 4D of CS but these were co-localized on the chromosomes of 4D in Jagger and TAM11, indicating the centromere repositioning in 4D of CS. In 4D of CS, pAs1 localization pattern tend to be positioned toward log arm and which is completely overlapped with CENH3. In Jagger and TAM111, however it was positioned toward to the short arm. The pSc119 was used for additional FISH marker to identify the chromosomes 4D in CS, Jagger and TAM111. D, Ideogram depicting distribution of each probe on the chromosomes of 4D in three wheat cultivars. (TIF) [file pone.0137747.s001.tif]

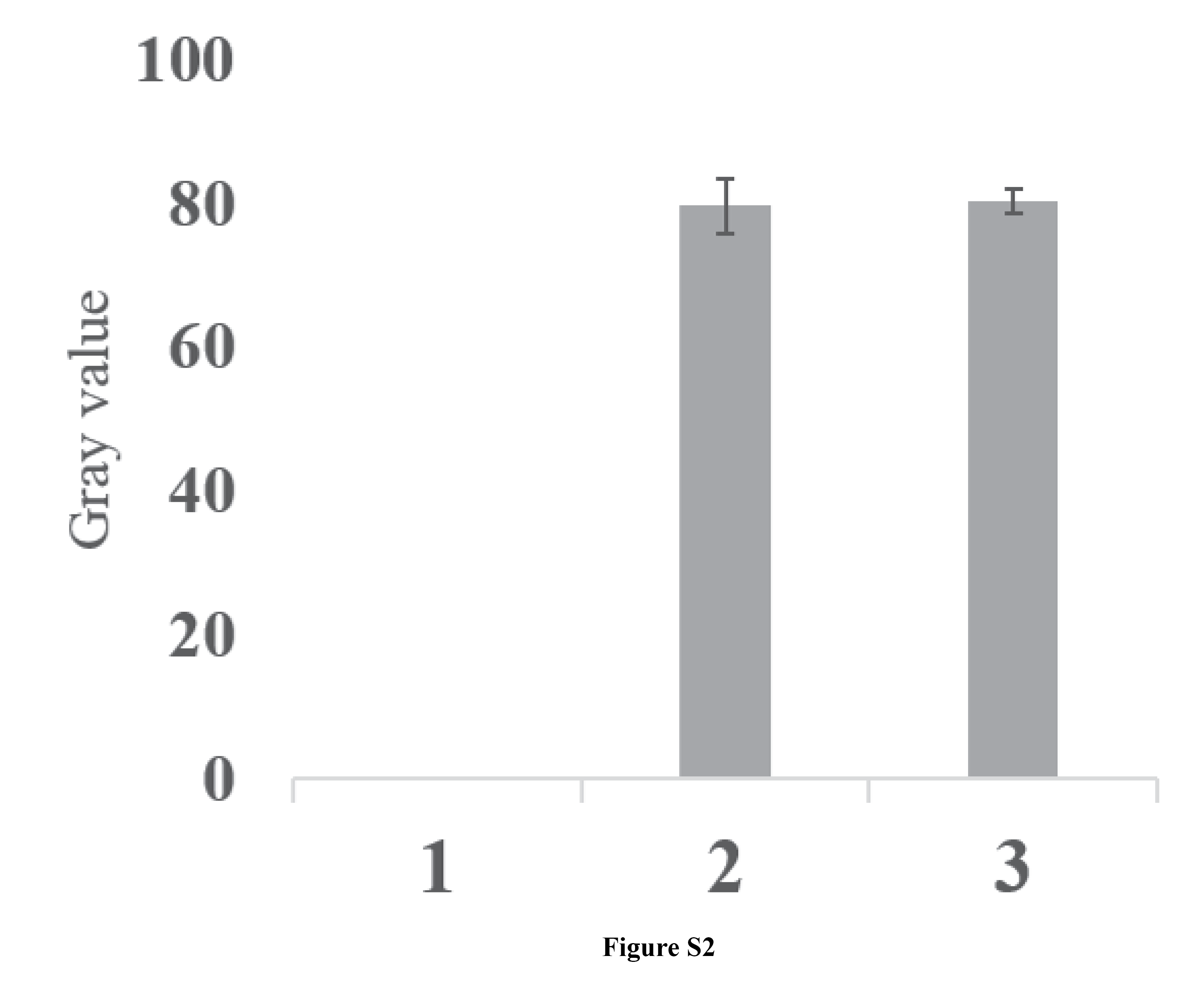

Supplement: S2 Fig — Numbers at y axis represent the gray value (relative signal intensity of antibody to background, background was normalized as zero). 1: background signal, 2: CENH3 signal intensity in dDt4DS, 3: CENH3 signal intensity in dDt4DL. Measurements were done by Image J software. The gray value of CENH3 was 79.6±3.8 (n = 4) and 80.2±1.7 (n = 4) in dDt4DS and dDt4DL, respectively. (TIF) [file pone.0137747.s002.tif]

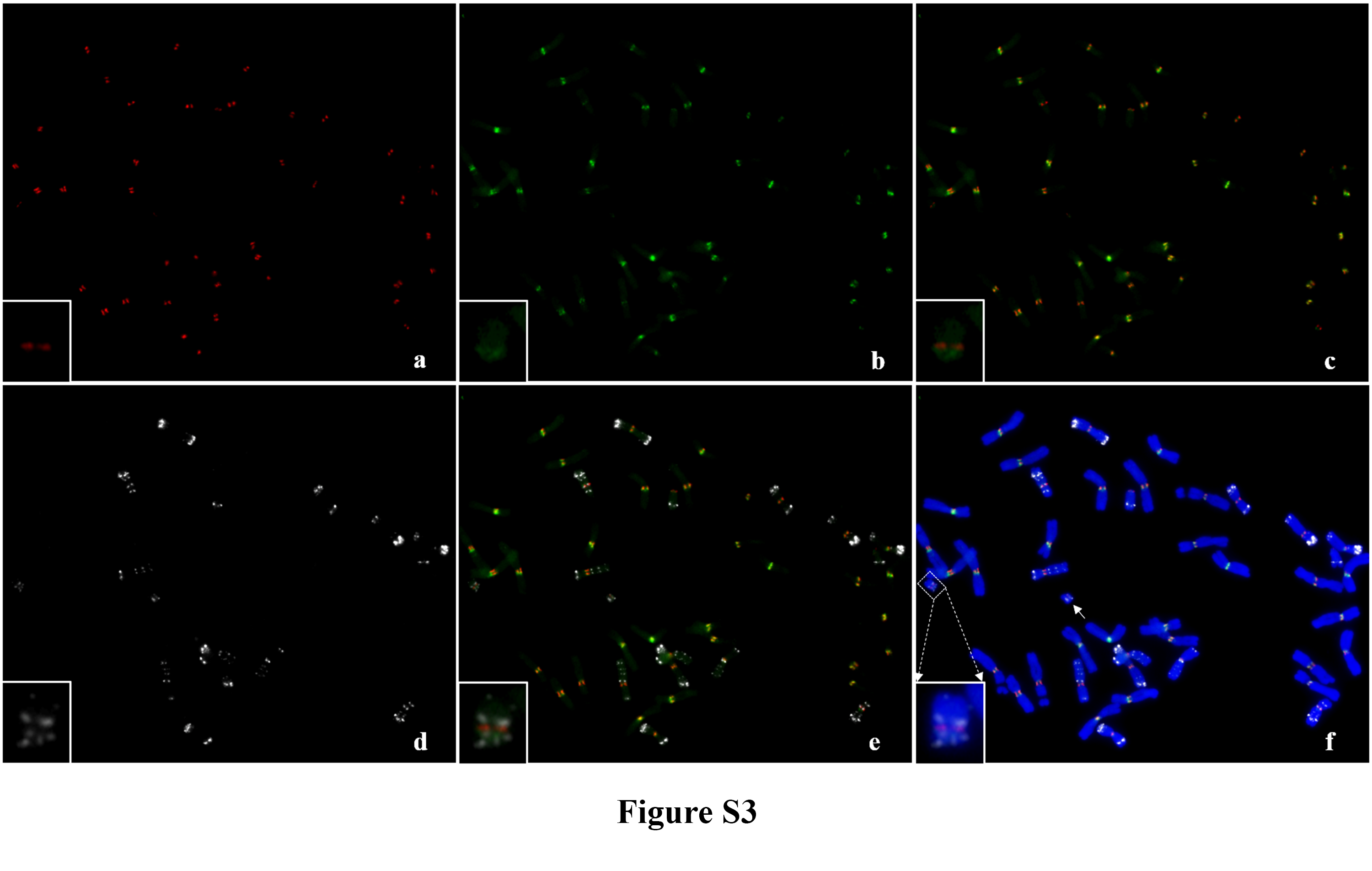

Supplement: S3 Fig — Merged images, CENH3 and CRWs (c), and CENH3, CRWs and pAs1 (e) with DAPI stained metaphase chromosome (f). The inserts show telosome, dDt1DS probed with CENH3 (red), CRWs (green) and pAs1 (white). CENH3 was detected by rhodamine-conjugated anti-rabbit antibodies (red), and the signals were fixed with 4% paraformaldehyde. The same metaphase cell was probed with CRWs (green) and pAs1 (far red, the signals were pseudocolored in white). (TIF) [file pone.0137747.s003.tif]

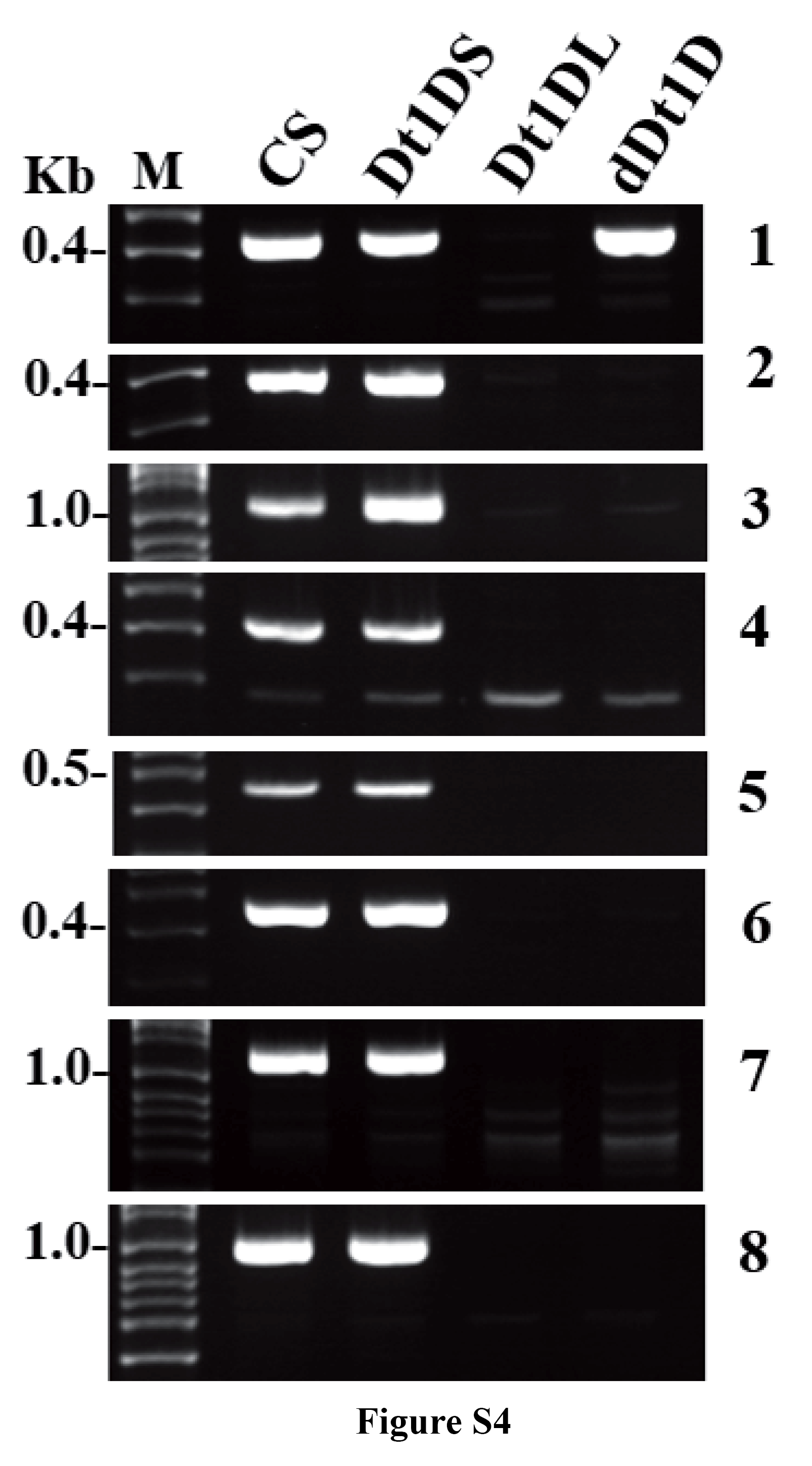

Supplement: S4 Fig — (TIF) [file pone.0137747.s004.tif]

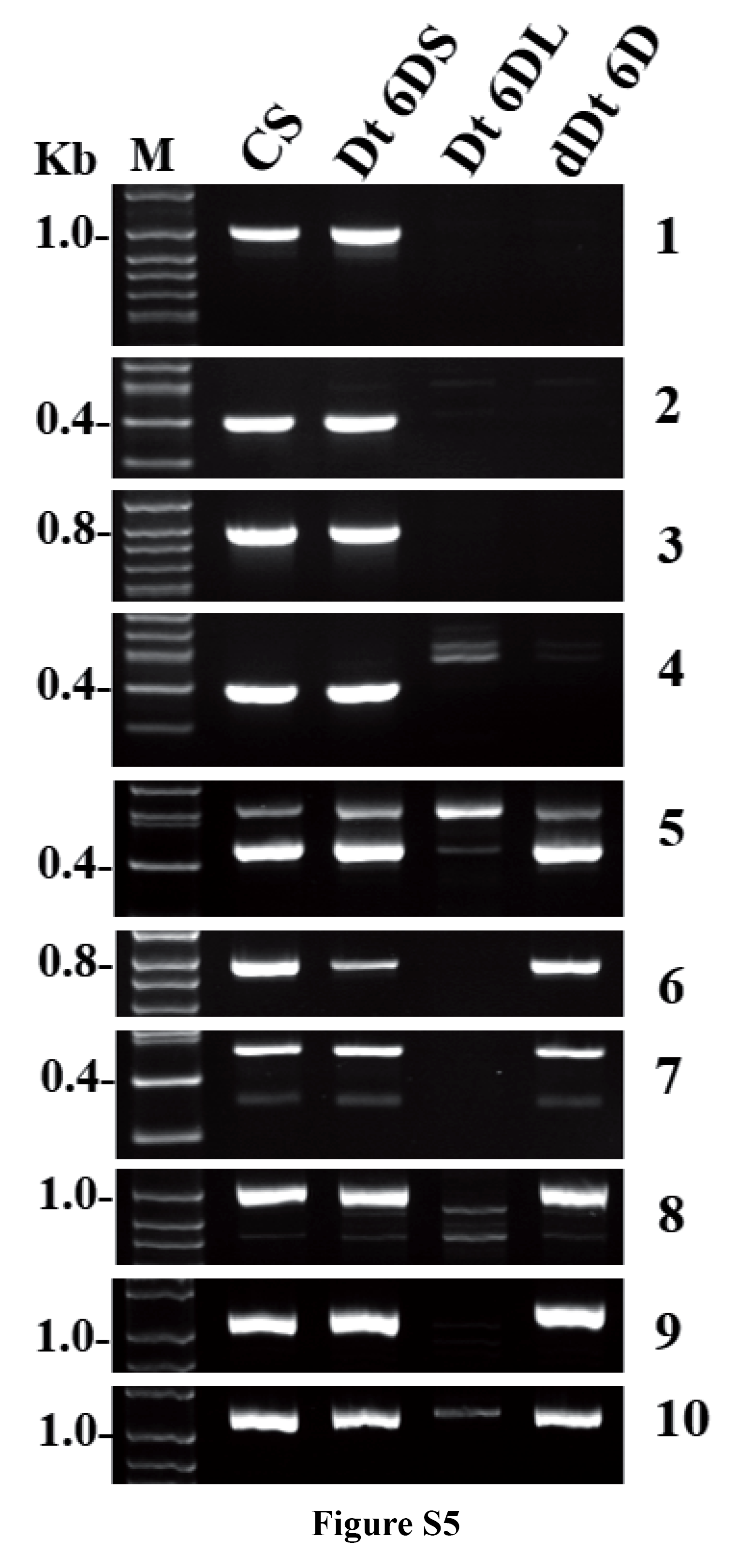

Supplement: S5 Fig — Four primers derived from terminal deletion bin had no amplification (1–4) while six primers derived from interstitial (5–8) and proximal deletion bin (9–10) had amplification in dDt6DS indicating terminal deletion in dDt6DS. (TIF) [file pone.0137747.s005.tif]

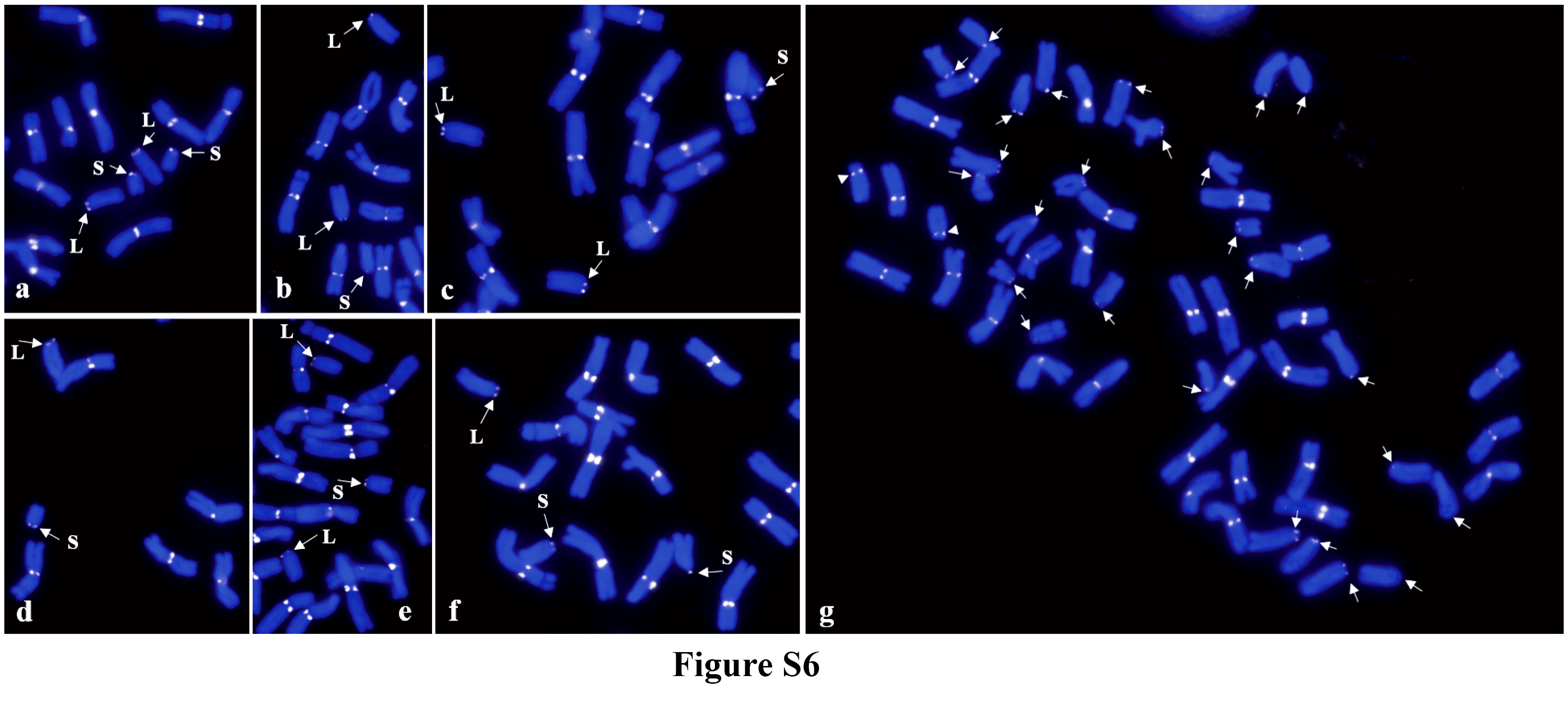

Supplement: S6 Fig — Telosomes are indicated by arrows. Immunofluorescence of CENH3 on CS containing 28 telosomes (g). The CENH3 fluorescent signals on 24 telosomes (derived from B-genome chromosomes are indicated by arrows) + one pair of t4AL were smaller than those of other intact chromosomes except 4AS (arrowhead) which is an acrocentric chromosome. (TIF) [file pone.0137747.s006.tif]
